# Hepatoduodenal ligament teratoma presenting with fever in a young adult

**DOI:** 10.1093/jscr/rjaf1056

**Published:** 2026-01-08

**Authors:** Mete Ucdal, Evren Ekingen

**Affiliations:** Etimesgut Sehit Sait Erturk State Hospital, Ankara, Turkiye; Department of Internal Medicine, Hacettepe University Faculty of Medicine, Ankara, Türkiye; Etimesgut Sehit Sait Erturk State Hospital, Ankara, Turkiye

**Keywords:** hepatoduodenal ligament, teratoma, fever, cholangitis, young adult, extragonadal germ cell tumor

## Abstract

Hepatoduodenal ligament teratomas are very rare, and there are fewer than 20 cases reported in the literature. A 22-year-old woman with fever and acute abdominal pain due a mature cystic teratoma in the hepatoduodenal ligament. This occurrence when presented with acute cholangitis was the first case of this rare tumor. Previous cases seen in adults were asymptomatic and were diagnosed incidentally on investigations. The mass was successfully removed operation on this patient without affecting the portal triad structures. This case adds to our knowledge of how these rare tumors may present clinically. It also highlights the relevance of this diagnosis in young adults with acute febrile illness and appropriate imaging characteristics.

## Introduction

Teratomas arise from pluripotent germ cells and contain tissue from all three embryonic layers: ectoderm, mesoderm, and endoderm. While most develop in gonads, extragonadal sites include the mediastinum, retroperitoneum, and sacrococcygeal region [1]. Hepatoduodenal ligament teratomas are extremely rare, with fewer than 20 cases in literature [2, 3]. Most occur in childhood, often diagnosed antenatally or presenting with abdominal distension [2]. Adult cases are exceedingly rare, typically incidental findings during imaging [4, 5]. The hepatoduodenal ligament extends from the porta hepatis to the duodenum, containing the portal triad: portal vein, hepatic artery, and common bile duct [6]. Teratomas at this site result from blocked germ cell migration during embryologic development. Proximity to portal triad structures creates technical challenges during resection due to potential iatrogenic injury [7]. We report a hepatoduodenal ligament teratoma in a 22-year-old woman presenting with fever and acute abdominal pain, not previously described.

## Case report

A healthy 22-year-old woman presented with right upper quadrant pain and fever for 3–4 days. She reported home temperature of 38.5°C and denied jaundice, nausea, vomiting, or weight loss. Examination revealed temperature 38.2°C, pulse 96/minute, blood pressure 120/75 mmHg, marked right upper quadrant tenderness with positive Murphy’s sign and peritoneal irritation. No mass or jaundice was detected.

Laboratory studies showed leukocytosis (12 500/μl) with neutrophilia. Liver enzymes showed mild alkaline phosphatase (185 U/L) and gamma-glutamyl transferase (95 U/L) elevation. Total bilirubin, aminotransferases, renal function, and alpha-fetoprotein were normal. Acute cholangitis or cholecystitis was suspected. She received intravenous ceftriaxone, metronidazole, and fluids.

**Figure 1:**
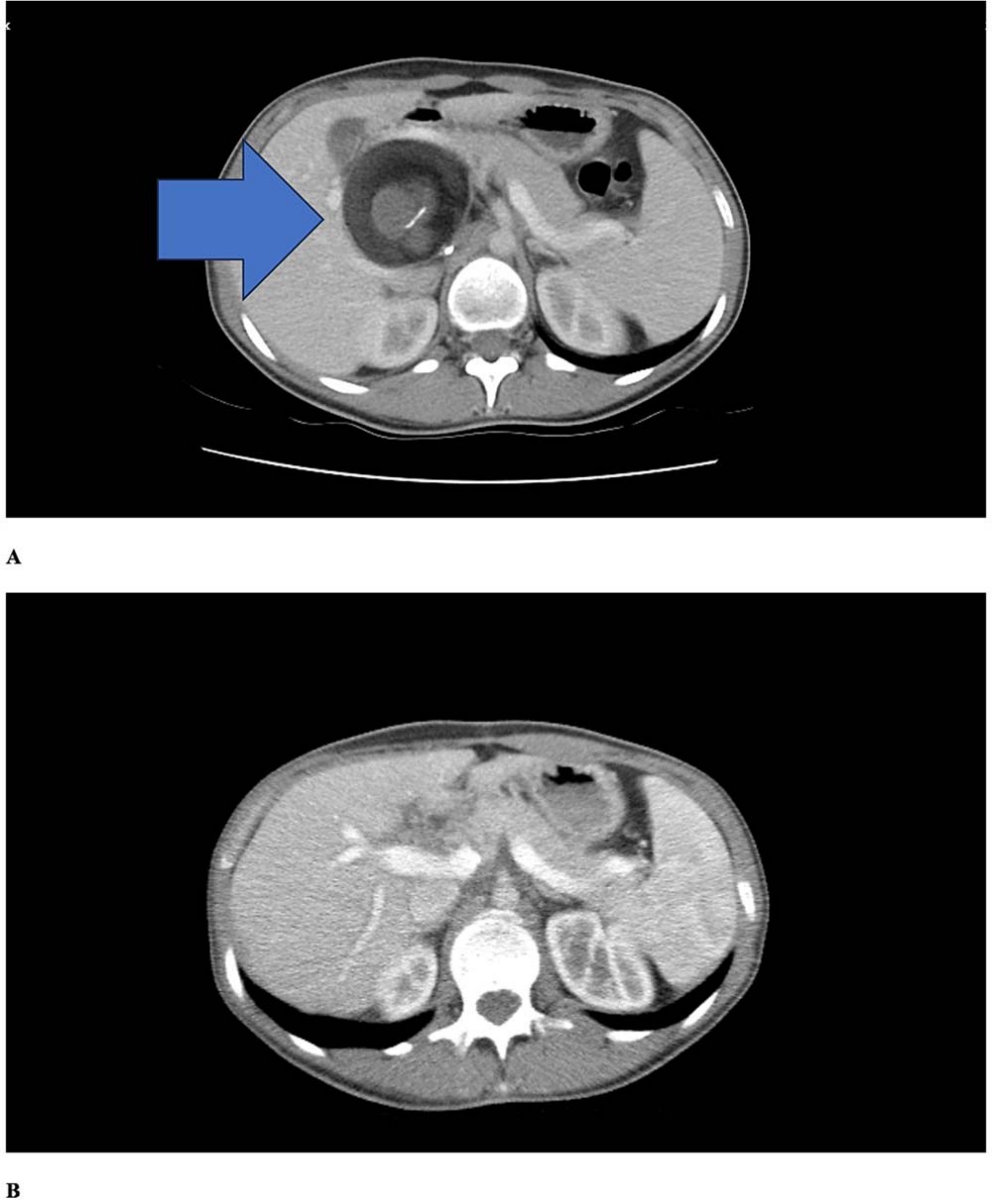
Contrast-enhanced axial CT scans. (a) Preoperative imaging showing a well-defined cystic lesion in the hepatoduodenal ligament region with heterogeneous attenuation including areas of fat density (negative Hounsfield units) and fluid components. (b) Six-month postoperative follow-up demonstrating complete resolution of the lesion with no evidence of recurrence. Periportal millimetric metallic densities representing surgical clips are visible.

Abdominal computed tomography (CT) demonstrated a 4–5 cm cystic lesion in the hepatoduodenal ligament (Fig. 1a) containing fat density and fluid without calcifications. No biliary dilatation, cholecystitis, or vascular compression was seen. The gallbladder was normal. The liver was slightly enlarged with normal contours. Minimal intrahepatic bile duct prominence was noted.

Fat attenuation within a hepatoduodenal cystic lesion suggested mature cystic teratoma, possibly infected given acute febrile presentation. After stabilization, semi-urgent surgery was planned. Fever resolved within 24 h with antibiotics, but surgery remained necessary.

At laparotomy, a 5 cm encapsulated cystic mass arose from the hepatoduodenal ligament between porta hepatis and duodenum. Inflammatory reaction was consistent with acute presentation. Portal triad structures were displaced but not encased. The tumor was carefully dissected, preserving portal vein, hepatic artery, and common bile duct. Complete excision was achieved without injury.

Gross examination revealed sebaceous material and hair. Histopathology confirmed mature cystic teratoma with well-differentiated tissues from all three germ layers: stratified squamous epithelium with dermal appendages (ectoderm), adipose tissue and cartilage (mesoderm), and respiratory epithelium (endoderm). No immature or malignant components were found.

Postoperative course was uncomplicated. Intravenous antibiotics continued 48 h, then oral antibiotics for 7 days total. She was discharged on Day 4. At six-month follow-up, she remained asymptomatic with normal alpha-fetoprotein. Follow-up CT (Fig. 1b) showed complete resolution without recurrence, normal liver and bile ducts, and surgical clips in the periportal region.

## Discussion

Most hepatoduodenal ligament teratomas occur in children (71%), presenting as asymptomatic mass, jaundice, or portal hypertension [8, 9]. Adult cases are quite rare, and most are asymptomatic and discovered incidentally [10]. Our case presented uniquely with fever (38.2°C), acute pain, and cholestasis, which had not been previously reported among the 16 cases in the literature (Table 1).

**Table 1:** Summary of all hepatoduodenal ligament teratoma cases reported in the literature to date, comparing demographic features, clinical presentation, tumor characteristics, surgical management, complications, pathology findings, follow-up duration, and outcomes

Case	Author, Year	Age/Sex	Presentation	Size	AFP	Surgery	Complication	Pathology	Immature	Follow-up	Outcome	Ref
1	Frexes, 1986	Newborn/−	Jaundice	Small		Local excision	Recurrence	Teratoma		5 years	Asymptomatic	[1]
2	Akimov, 1989	6 yr/−	Portal HTN				Fatal				Death	[2]
3	Kim, 1992	5 yr/M	Jaundice			Whipple	Fatal	Yolk sac + teratoma	Yes		Death	[4]
4	Demircan, 2004	4 mo/F	Jaundice	15 cm	Normal	Excision + reconstruction	None	Mature	No	4 years	Good	[5]
5	Wang, 2004	29 yr/F	Portal HTN	7 × 6 × 6	Normal	Extirpation	None	Mature	No	2 years	Good	[6]
6	Sasaki, 2005	38 yr/M	Mass	8 cm		Excision + choledochojejunostomy	None	Mature cystic	No		Good	[9]
7	Ukiyama, 2008	20 mo/M	Mass	9 × 6 × 6	2.3	Extirpation	PV laceration	Mature	No	5 years	Good	[7]
8	Brown, 2008	1–2 mo/F	Asymptomatic			Local excision	None	Mature	No		Good	[3]
9	Bagga, 2012	11 yr/F	Jaundice	9 × 9 cm	Normal	Lilly technique	PV laceration	Mature cystic	No	2 years	Good	[11]
10	Ohno, 2013	3 mo/F	Mass	13.2 cm	Normal	Local excision	None	Immature	Yes		Good	[12]
11	Jeismann, 2014	27 yr/F	Asymptomatic	5 × 4 cm	Normal	Local excision	None	Mature	No	6 months	Good	[10]
12	De Roo, 2017	Newborn/F	Mass	10.6 cm	212 340	Local excision	None	Immature+yolk sac	Yes	7 months	No recurrence	[16]
13	Gundapaneni, 2018	2 yr/F	Mass	10 × 8 × 7	Normal	Extirpation	None	Mature	No	6 months	Good	[13]
14	Gazula, 2020	5 mo/F	Distension	15 × 10	Normal	Excision + choledochojejunostomy	CBD injury	Mature (Grade 0)	No	30 months	No recurrence	[14]
15	Ravikumar, 2018	7 days/M	Mass	Large	20 000	Local excision	PV + HA + CBD transection	Immature (Grade III)	Yes	6 months	Good	[8]
16	Miyazaki, 2021	60 s/F	Asymptomatic	40 × 32 mm		Extirpation	None	Mature	No	2 yr 10 mo	No recurrence	[15]
17	Our case, 2025	24 yr/M	Fever + acute pain	6 × 5 cm	Normal	Extirpation	CBD fistulization	Mature	No	12 months	No recurrence	

M, male; F, female; HTN, hypertension; PV, portal vein; HA, hepatic artery; CBD, common bile duct; mo, months; yr, years; Ref, refer.

Acute febrile presentation likely resulted from secondary bacterial infection. Mature cystic teratomas contain sebaceous material, hair, and dental tissue, providing excellent bacterial growth medium [11]. Hepatoduodenal ligament proximity to duodenum increases gastrointestinal bacterial contamination risk. Intraoperatively, cyst fistulization to the common bile duct with hair extending into the bile duct was noted. This direct connection may have facilitated bacterial entry. Fever resolution within 24 h with antibiotics strongly supports secondary infection.

Differential diagnosis includes choledochal cyst, cholecystoduodenal fistula, ectopic thyroid, and hepatoid adenocarcinoma [3, 12]. Normal AFP and histopathology confirmed mature cystic teratoma without immature or malignant elements.

CT is critical for diagnosis. Heterogeneous masses with fat density, calcifications, and cystic components strongly suggest teratoma [13]. Normal AFP levels favor mature teratoma.

Surgical management is challenging due to portal triad proximity, significantly increasing injury risk [14]. Ukiyama *et al*. [5] emphasized intraoperative ultrasonography and Doppler utility. Bagga *et al*. [11] experienced portal vein laceration despite using the Lilly technique. Ravikumar *et al*. [14] reported complete transection of portal vein, hepatic artery, and common bile duct, requiring repair and 350 ml transfusion.

In our case, anatomical relationships were mapped with preoperative high-resolution CT, and the tumor was excised without damage to vascular structures through meticulous dissection. Because the common bile duct was fistulized, excision included gallbladder, and distal common bile duct. With portal vein and hepatic artery posteriorly located, the Lilly technique was applied. Hepaticoduodenostomy established bilioenteric continuity. No complications occurred; at 2-year follow-up, the patient remained asymptomatic without recurrence.

Histopathologically, 76% of cases are mature teratomas, 18% immature, and 6% contain malignant components. Only three cases received chemotherapy [12, 15, 16].

## Conclusion

Hepatoduodenal ligament teratomas are extremely rare. This is the first reported acute febrile case. In young adults with acute abdominal pain and cholestasis, extragonadal germ cell tumors should be considered, particularly for cystic lesions fistulized to the common bile duct. CT and AFP evaluation are critical for diagnosis. Due to portal triad proximity, resection requires preoperative anatomical planning and surgical expertise. Prognosis after complete excision is excellent.
